# The natural history of classic galactosemia: lessons from the GalNet registry

**DOI:** 10.1186/s13023-019-1047-z

**Published:** 2019-04-27

**Authors:** M. E. Rubio-Gozalbo, M. Haskovic, A. M. Bosch, B. Burnyte, A. I. Coelho, D. Cassiman, M. L. Couce, C. Dawson, D. Demirbas, T. Derks, F. Eyskens, M. T. Forga, S. Grunewald, J. Häberle, M. Hochuli, A. Hubert, H. H. Huidekoper, P. Janeiro, J. Kotzka, I. Knerr, P. Labrune, Y. E. Landau, J. G. Langendonk, D. Möslinger, D. Müller-Wieland, E. Murphy, K. Õunap, D. Ramadza, I. A. Rivera, S. Scholl-Buergi, K. M. Stepien, A. Thijs, C. Tran, R. Vara, G. Visser, R. Vos, M. de Vries, S. E. Waisbren, M. M. Welsink-Karssies, S. B. Wortmann, M. Gautschi, E. P. Treacy, G. T. Berry

**Affiliations:** 1 Department of Pediatrics and Clinical Genetics, GROW-School for Oncology and Developmental Biology, Maastricht University Medical Centre, P. Debyelaan 25, P.O. Box 5800, 6202 AZ Maastricht, The Netherlands; 2 Amsterdam UMC, University of Amsterdam, Pediatric Metabolic Diseases, Emma Children’s Hospital, Amsterdam, Netherlands; 3 Institute of Biomedical Sciences of the Faculty of Medicine of Vilnius University, Vilnius, Lithuania; 4 Metabolic Center, Department of Gastroenterology-Hepatology, Leuven University Hospitals and KU Leuven, Leuven, Belgium; 5 Unit of Diagnosis and Treatment of Congenital Metabolic Diseases, S. Neonatology, Department of Pediatrics, Hospital Clínico Universitario de Santiago de Compostela, CIBERER, Health Research Institute of Santiago de Compostela (IDIS), Santiago de Compostela, Spain; 6 Department of Endocrinology, Queen Elizabeth Hospital Birmingham, London, UK; 7 Manton Center for Orphan Disease Research, Division of Genetics and Genomics, Boston Children’s Hospital, Harvard Medical School, Boston, MA USA; 8 Section of Metabolic Diseases, Beatrix Children’s Hospital, and Groningen University Institute for Drug Exploration (GUIDE), University Medical Center Groningen, University of Groningen, Groningen, The Netherlands; 9 Antwerp University Hospital, Antwerp, Belgium; 10 Hospital Clinic Barcelona, Barcelona, Spain; 11 Metabolic Medicine Department, Great Ormond Street Hospital, Institute for Child Health UCL, London, UK; 12 Division of Metabolism and Children’s Research Center, University Children’s Hospital, Zurich, Switzerland; 13 Department of Endocrinology, Diabetes, and Clinical Nutrition, University Hospital Zurich, Zurich, Switzerland; 14 APHP, HUPS, Hôpital Antoine Béclère, Centre de Référence Maladies Héréditaires Hépatiques, Clamart, France; 15 Université Paris Sud-Paris Saclay, and INSERM U 1195, Paris, France; 16 Department of Pediatrics, Center for Lysosomal and Metabolic Diseases, Erasmus MC-Sophia Children’s Hospital, Rotterdam, The Netherlands; 17 Department of Pediatrics, Hospital Santa Maria, Centro Hospitalar Universitário Lisboa Norte EPE, Lisbon, Portugal; 18 Institute for Clinical Biochemistry and Pathobiochemistry, German Diabetes Center, Leibniz Center for Diabetes Research at Heinrich Heine University, Düsseldorf, Germany; 19 National Centre for Inherited Metabolic Disorders, Temple Street Children’s University Hospital, Temple Street, Dublin, Ireland; 20 Metabolic Disease Unit, Edmond and Lily Safra Children’s Hospital, Sheba Medical Center, and Sackler Faculty of Medicine, Tel Aviv University, Tel Aviv, Israel; 21 Department of Internal Medicine, Center for Lysosomal and Metabolic Diseases, Erasmus MC, University Medical Center Rotterdam, Rotterdam, The Netherlands; 22 Department for Pediatrics and Adolescent Medicine, Inborn Errors of Metabolism, Medical University of Vienna, Vienna, Austria; 23 Clinical Research Center, Department of Medicine I, University Hospital RWTH Aachen, Aachen, Germany; 24 Charles Dent Metabolic Unit, National Hospital for Neurology and Neurosurgery, London, UK; 25 Department of Clinical Genetics, United Laboratories and Institute of Clinical Medicine, Tartu University Hospital, Tartu, Estonia; 26 Department of Pediatrics, University Hospital Centre, Zagreb, Croatia; 27 Research Institute for Medicines (iMed.ULisboa), and Department of Biochemistry and Human Biology, Faculty of Pharmacy, Universidade de Lisboa, Lisbon, Portugal; 28 Universitätsklink für Pädiatrie, Tirol Kliniken GmbH, Innsbruck, Austria; 29 Mark Holland Metabolic Unit, Adult Inherited Metabolic Disorders Department, Salford Royal NHS Foundation Trust, Salford, M6 8HD UK; 30 Vrije Universiteit Amsterdam, Internal Medicine, Amsterdam UMC, Amsterdam, Netherlands; 31 Center for Molecular Diseases, Division of Genetic Medicine, University Hospital Lausanne, Lausanne, Switzerland; 32 Department of Paediatric Inherited Metabolic Disease, Evelina London Children’s Hospital, London, UK; 33 Department of Pediatrics, University Medical Centre Utrecht, Utrecht, The Netherlands; 34 Department of Methodology and Statistics, CAPHRI School for Primary Care and Public Health, Faculty Health Medicine and Life Sciences, Maastricht, The Netherlands; 35 Department of Pediatrics, Radboud University Medical Center, Nijmegen, The Netherlands; 36 Department of Pediatrics, Division of Genomics and Genetics, Harvard Medical School and Boston Children’s Hospital, Boston, USA; 37 University Children’s Hospital, Parcelsus Medical University (PMU), Salzburg, Austria; 38 Department of Pediatrics and Institute of Clinical Chemistry, Inselspital, University Hospital Bern, Bern, Switzerland; 39 National Centre for Inherited Metabolic Disorders, Mater Misericordiae University Hospital, Dublin 7, Ireland

**Keywords:** Registry, Natural history, Galactosemia, GALT deficiency, Galactosemia network

## Abstract

**Background:**

Classic galactosemia is a rare inborn error of carbohydrate metabolism, caused by a severe deficiency of the enzyme galactose-1-phosphate uridylyltransferase (GALT). A galactose-restricted diet has proven to be very effective to treat the neonatal life-threatening manifestations and has been the cornerstone of treatment for this severe disease. However, burdensome complications occur despite a lifelong diet. For rare diseases, a patient disease specific registry is fundamental to monitor the lifespan pathology and to evaluate the safety and efficacy of potential therapies. In 2014, the international Galactosemias Network (GalNet) developed a web-based patient registry for this disease, the GalNet Registry. The aim was to delineate the natural history of classic galactosemia based on a large dataset of patients.

**Methods:**

Observational data derived from 15 countries and 32 centers including 509 patients were acquired between December 2014 and July 2018.

**Results:**

Most affected patients experienced neonatal manifestations (79.8%) and despite following a diet developed brain impairments (85.0%), primary ovarian insufficiency (79.7%) and a diminished bone mineral density (26.5%). Newborn screening, age at onset of dietary treatment, strictness of the galactose-restricted diet, p.Gln188Arg mutation and GALT enzyme activity influenced the clinical picture. Detection by newborn screening and commencement of diet in the first week of life were associated with a more favorable outcome. A homozygous p.Gln188Arg mutation, GALT enzyme activity of ≤ 1% and strict galactose restriction were associated with a less favorable outcome.

**Conclusion:**

This study describes the natural history of classic galactosemia based on the hitherto largest data set.

**Electronic supplementary material:**

The online version of this article (10.1186/s13023-019-1047-z) contains supplementary material, which is available to authorized users.

## Background

Classic galactosemia (CG, OMIM # 230400) is a rare inborn error of carbohydrate metabolism, caused by a severe deficiency of the enzyme galactose-1-phosphate uridylyltransferase (GALT, E.C. 2.7.7.12). GALT is the second enzyme in the Leloir pathway, the main route of galactose metabolism. CG has a prevalence in western countries of between 1:16,000 and 1:60,000 live births [1, 2]. At present, over 300 variations in the *GALT* gene have been identified, with c.563A>G (p.Gln188Arg) being the most common pathogenic variation among people of European ancestry [3].

The first description of a neonate with galactosemia showing acute systemic toxicity was in 1908. In 1935, the case of an infant with hypergalactosemia and galactosuria who responded well to a lactose-restricted diet at 10 months of age was described [4]. In 1956, GALT was characterized as the enzyme that is affected in CG [5] and in 1988 the *GALT* gene was identified [6]. The pathophysiology is complex and not fully understood. Various mechanisms have been implicated [7–16].

CG presents in the neonatal period when upon exposure to galactose-containing milk, newborns develop feeding difficulties, failure to thrive, hepatocellular damage, *E. coli* sepsis, hypotonia, renal tubular disease and cataracts [13]. Several countries have implemented newborn screening (NBS) for CG. The current standard of care, a galactose-restricted diet, resolves the neonatal clinical picture. Unfortunately, despite diet, most patients develop complications that affect mainly the central nervous system and the female gonads, resulting in cognitive, neurological and behavioral complications and primary ovarian insufficiency (POI) with subsequent subfertility in female patients [17, 18]. In addition, patients are at risk of a diminished bone mineral density (BMD) [19, 20]. The clinical phenotype can vary considerably even in patients harboring the same genotype and within the same family.

The scarcity of relevant knowledge and outcome experience with most rare diseases creates a need for disease specific patient registries. Rare disease registries are a tool to gather comprehensive knowledge to improve patient care, to monitor the pathogenesis of a disorder over a lifespan and to support clinical research, particularly the safety and efficacy evaluation of potential therapies and treatment strategies [21–23].

The international network for the galactosemias (GalNet) [24] developed and implemented a web-based patient registry in 2014, the GalNet registry, that includes type I (classic and variant galactosemia), type II (galactokinase deficiency) and type III galactosemia (galactose epimerase deficiency). This study aims to delineate the natural history of classic galactosemia based on a large data set of patients. This information is of utmost importance for all stakeholders involved in the care of this group of patients.

## Results

### Patients’ characteristics

A total of 509 patients (48.1% male and 51.9% female) from 15 countries were included; data was collected from December 2014 to July 2018. The age ranged from 0 to 65 years (median 18.0 years) and the majority of patients were Caucasian, 93.6% (436/466). Mutational analysis revealed c.563A > G (p.Gln188Arg) homozygosity as the most common genotype, in 57.7% (233/404). Because of the coded character of the data, we have no information on sibling relationship to elaborate on the number of independent mutant alleles. Enzyme activity was ≤ 1% in 82.7% (211/255) of patients. Diagnosis was established following a positive newborn screening (NBS) in 45.9% (215/468) of patients (Table 1).

**Table 1:** Patients’ characteristics

	n	Valid n	%
Gender		509	
Male	245		48.1
Female	264		51.9
Age (years)		509	
< 18 years	233		45.8
≥ 18 years	276		54.2
Ethnicity		466	
Caucasian	436		93.6
Other^a^	30		6.4
*GALT* gene mutation^b^		404	
c.[563A>G];[563A>G] (p.[(Gln188Arg)];[(Gln188Arg)])	233		57.7
c.[563A>G];[855G>T] (p.[(Gln188Arg)];[(Lys285Asn)])	29		7.2
c.[563A>G];[584T>C] (p.[(Gln188Arg)];[(Leu195Pro)])	10		2.5
c.[563A>G];[5.2 kb del]	5		1.2
c.[855G>T];[855G>T] (p.[(Lys285Asn)];[(Lys285Asn)])	7		1.7
c.[855G>T];[584T>C] (p.[(Lys285Asn)];[(Leu195Pro)])	2		0.5
c.[584T>C];[584T>C] (p.[(Leu195Pro)];[(Leu195Pro)])	3		0.7
c.[5.2 kb del];[5.2 kb del]	3		0.7
Other	112		27.7
Enzyme activity		255	
≤ 1%	211		82.7
> 1 ≤ 5%	36		14.1
> 5 ≤ 10%	8		3.1
Diagnosed following NBS	215	468	45.9

Median age 18 years, range 0–67 years

^a^Black, Mixed, Asian, North African

^b^For simplicity reasons, the mutation NM_000155.2(GALT):c.[−1039_753del;820 + 50_*789delinsGAATAGACCCCA] is here mentioned as 5.2 kb del

### Neonatal illness

Neonatal illness was reported in 79.8% (332/416) of patients. The commonest documented abnormalities were elevated liver enzymes in 70.3% (211/300), bleeding diathesis in 42.5% (128/301), encephalopathy in 29.0% (71/245), clinical signs of infection in 27.4% (96/351), cataract in 25.8% (68/264) and hypoglycemia in 25.1% (65/259). The neonatal erythrocyte galactose-1-phosphate (Gal-1-P) peak level was increased in 90.8% (89/98) of patients (Table 2). Diagnosis following NBS and early initiation of galactose restriction within the first week of life were associated with a lower odds ratio for neonatal complications (*p* < 0.0000001; OR 0.30 [0.20–0.47] and *p* < 0.000001; OR 0.32 [0.21–0.50], respectively). Patients diagnosed following the NBS were often younger (*p* < 0.000001) and started diet in the first week of life (*p* < 0.000000000001). An enzyme activity of ≤ 1% was associated with a higher rate of acute neonatal illness (*p* = 0.017; OR 2.65 [1.23–5.70]).

**Table 2:** Neonatal illness

	n	Valid n	%
Acute neonatal illness^a^	332	416	79.8
Elevated liver enzymes (ALT, AST > 30 U/L)	211	300	70.3
Bleeding diathesis (abnormal PT/ APTT)	128	301	42.5
Encephalopathy^b^	71	245	29.0
Signs of infection	96	351	27.4
Positive blood culture	36	64	56.3
Cataract	68	264	25.8
Hypoglycemia (< 2.6 mmol/L)	65	259	25.1
Increased neonatal Gal-1-P (> 0.05 μmol/g Hb or > 10 mg/dL)	89	98	90.8

^a^Defined as having one of the following symptoms: encephalopathy, bleeding diathesis, signs of infection, elevated liver enzymes or hypoglycemia

^b^Altered mental state: depressed consciousness with or without neurological signs

### Long-term complications

#### Neurological, cognitive and behavioral complications

**Fig. 1:**
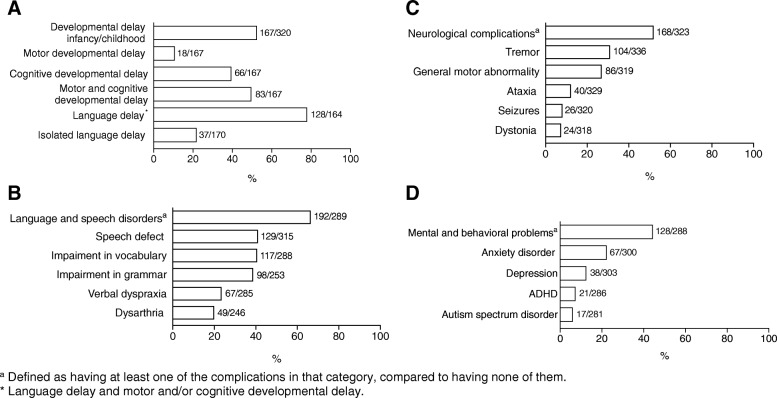
Frequency of neurological, cognitive and mental (psychiatric) complications. **a** Developmental delay infancy/childhood. **b** Language and speech disorders. **c** Neurological complications. **d** Mental (psychiatric) and behavioral problems. The n/valid n is shown per outcome

Brain impairments were frequently reported, in 85.0% (277/326) of patients (Table 3, Fig. 1). Global developmental delay was documented in 52.2% (167/320) of the patients. A great majority in this group, 78.0% (128/164) of patients, showed also a language delay. Additionally, isolated language delay was reported for 21.8% (37/170) of the patients. No gender differences were observed.

**Table 3:** Neurological, cognitive and mental (psychiatric) complications

	n	Valid n	%
Developmental delay infancy/childhood	167	320	52.2
Motor	18		10.8
Cognitive	66		39.5
Motor and cognitive	83		49.7
Language delay^b^	128	164	78.0
Isolated language delay	37	170	21.8
Language and speech disorders^a^	192	289	66.4
Speech defect	129	315	41.0
Impairment in vocabulary	117	288	40.6
Impairment in grammar	98	253	38.7
Verbal dyspraxia	67	285	23.5
Dysarthria	49	246	19.9
Neurological complications^a^	168	323	52.0
Tremor	104	336	31.0
General motor abnormality	86	319	27.0
Ataxia	40	329	12.2
Seizures	26	320	8.1
Dystonia	24	318	7.5
Mental (psychiatric) and behavioral problems^a^	128	288	44.4
Anxiety disorder	67	300	22.3
Depression	38	303	12.5
ADHD	21	286	7.3
Autism spectrum disorder	17	281	6.0

^a^Defined as having at least one of the complications in that category, compared to having none of them

^b^Language delay and motor and/or cognitive developmental delay

Language and speech disorders were often reported, in 66.4% (192/289) of the patients, with speech defect in 41.0% (129/315), impairment in vocabulary in 40.6% (117/288), impairment in grammar in 38.7% (98/253), verbal dyspraxia in 23.5% (67/285), and dysarthria in 19.9% (49/246) of patients. Language and speech disorders were more often reported in young male patients (*p* = 0.034).

Analysis of neurological complications data revealed a prevalence of 52.0% (168/323) in the study population, with tremor as most frequent complication in 31.0% (104/336) of the patients. Tremor was more often first detected after the second decade of life, in 41.3% (26/63) of patients, but also between pre-school age and the second decade, in 34.9% (22/63) of patients and between the first year and pre-school age, in 23.8% (15/63) of patients. Other neurological complications were general motor abnormality (clumsiness, coordination difficulties) in 27.0% (86/319), ataxia in 12.2% (40/329), seizures in 8.1% (26/320) and dystonia in 7.5% (24/318) of patients. Some patients exhibited a combination of the above-mentioned neurological complications. In none of the patients, chorea or athetosis was reported. General motor abnormality was reported most frequently at pre-school age, whereas ataxia, seizures and dystonia manifested at all ages. Male and female patients were equally affected.

Mental and behavioral problems occurred in 128/288, 44.4% of the patients with a higher frequency in male patients as they grow older (*p* = 0.017). The most frequently reported were anxiety disorder in 22.3% (67/300) of patients. Other complications included depression, in 12.5% (38/303), ADHD in 7.3% (21/286) and ASD in 6.0% (17/281) of patients. The time of onset of mental and behavioral problems varied: depression was mainly seen after the second decade. Anxiety disorders were common in all age categories, with 36.8% (14/38) of patients presenting between pre-school age and the second decade and 55.3% (21/38) in the second or third decade of life. In 7.9% (3/38) of patients, anxiety disorders were reported in the pre-school age. ADHD and ASD were more likely to occur in early life, before the second decade.

Further analysis revealed that neurological complications were less prevalent in subjects with age below 18 years (*p* < 0.00000001; OR 0.15 [0.15–0.39]) and in patients diagnosed following NBS (*p* < 0.00001; 0.32 [0.20–0.51]). These patients more often were started on diet therapy in the first week of life (*p* < 0.000000000001), unlike those who were not diagnosed earlier following NBS. Patients with a strict diet (lactose restricted and restrictions in fruit and vegetables) developed neurological complications more frequently (*p* < 0.001; OR 2.81 [1.64–4.50]) than patients with a less strict diet.

Mental (psychiatric) and behavioral problems were less often reported in younger patients (*p* < 0.001); OR 0.42 [0.26–0.68]). An enzyme activity ≤ 1% was associated with a higher occurrence of mental and behavioral problems (*p* = 0.010; OR 3.41 [1.37–8.50]). Patients old enough to be assessed, more often did not reach a high level of education [25], 16.4% (29/177) compared to 30.7% (59/192) of the mothers and 42.7% (82/192) of the fathers (Additional file 1: Table S1). Patients attended special education programs more frequently, in 26.1% (42/161). The occupation [26] showed that patients perform unskilled occupations more often, in 45.6% (68/149) compared to their parents (16.5% (33/200) fathers and 26.8% (56/209) mothers) (Additional file 2: Table S2).

#### Gonadal complications

**Fig. 2:**
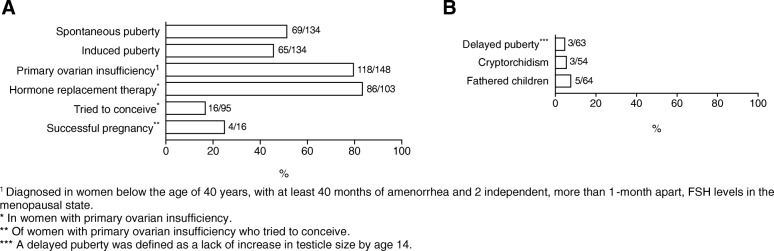
Frequency of gonadal complications. **a** Gonadal complications in female patients. **b** Gonadal complications in male patients. The n/valid n is shown per outcome

Spontaneous puberty was reported in 51.5% (69/134) of the female patients whereas 48.5% (65/134) had a delayed/induced puberty. The median age at spontaneous puberty was 13 years (range 10 to 17 years). The median age at induction of puberty was 13 (range 9 to 20 years). POI was reported in 79.7% (118/148) of female patients. In females aged > 35 years, POI percentage increased to 85.1% (40/47). In women with POI, 83.5% (86/103) patients reported to use hormone replacement therapy (HRT), median age of start of the HRT was 16 years (range 11 to 45 years). In the studied population, 16.8% (16/95) of female patients with POI tried to conceive and 25.0% (4/16) of these women successfully became pregnant without assisted reproduction. The median age of women when their first child was born was 25 years (range 17 to 38 years). Further analysis showed that a homozygous p.Gln188Arg mutation was associated with a higher odds ratio for POI (*p* = 0.040; OR 2.84 [1.08–7.47]). Delayed puberty in boys was reported in 4.8% (3/63) of patients. Male patients suffered from cryptorchidism in 5.6% (3/54) and 7.8% (5/64) had fathered a child (Table 4, Fig. 2).

**Table 4:** Gonadal complications

	n	Valid n	%
Female			
Puberty		134	
Spontaneous puberty	69		51.5
Induced puberty	65		48.5
Primary ovarian insufficiency^a^	118	148	79.7
Hormone replacement therapy	86	103	83.5
Tried to conceive	16	95	16.8
Successful pregnancy^b^	4	16	25.0
Male			
Delayed Puberty^c^	3	63	4.8
Cryptorchidism	3	54	5.6
Fathered children	5	64	7.8

^a^Diagnosed in women below the age of 40 years, with at least 40 months of amenorrhea and 2 independent, more than 1-month apart, FSH levels in the menopausal state

^b^Of women who tried to conceive

^c^A delayed puberty was defined as a lack of increase in testicle size by age 14

#### Bone health

**Fig. 3:**
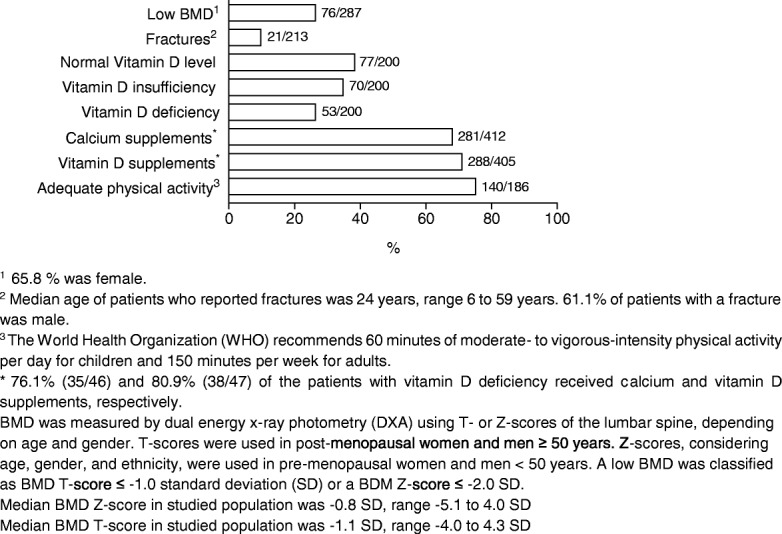
Frequency of outcomes in bone health. The n/valid n is shown per outcome

The median BMD Z-score of the studied population was − 0.8 SD (range − 5.1 to 4.0 SD), the median T-score was − 1.1 SD (range − 4 to 4.3 SD). A diminished BMD, defined as a BMD T-score ≤ − 1,0 standard deviation (SD) or a BDM Z-score ≤ − 2,0 SD, was reported in 26.5% (76/287) of the patients, where 65.8% (50/76) was female (Additional file 3: Table S3, Fig. 3). Fracture prevalence in this population was 9.9% (21/213). The median age of the patients with a fracture was 24 years (range 6 to 59 years). A low BMD was present in 23.8% (5/21) of the patients with fractures, 61.1% of patients with a fracture were male. Vitamin D deficiency (< 50 nmol/L) [27, 28] was documented in 26.5% (53/200). The majority of patients received calcium and vitamin D supplements (68.2% (281/412) and 71.1% (288/405), respectively). In the vitamin D deficiency group, 76.1% (35/46) and 80.9% (38/47) of the patients received calcium and vitamin D supplements, respectively. Physical activity, according to the World Health Organization (WHO) advice [29], was reported for 75.3% (140/186) of the patients. In 31/49 (63.3%) of patients with sufficient physical activity, a low BMD was reported. Patients with a low BMD were taking Vitamin D and calcium supplements in 94.1% (64/68) and 95.7% (67/70) respectively.

#### Cataract

Cataract in the neonatal period was reported in 25.8% (68/264) of the patients. In 54.5% (24/44) the cataract disappeared after introduction of diet, whereas in 45.5% (20/44) of patients a residual cataract was documented. A minority of patients developed cataract after the neonatal period, 9.2% (22/238). There was another group of patients, 11.2% (10/89), in whom cataract was reported in adulthood (median 29.5 years, range 18 to 41 years) (Additional file 4: Table S4). No information on gross deviations from diet or other reasons for cataract is available.

### Diet

During the neonatal period, most of the children were given a soy infant formula 76.6% (302/394). A minority, 12.7% (50/394), received elemental formula and the remainder had other galactose-restricted formulas, 10.7% (42/394). Diet was implemented within the first day of life in 16.6% (65/391) of the patients, whereas 33.9% (133/391) of the patients started diet on the remaining days of the first week of life. In 34.2% (134/391) of the cases diet was implemented in the second week, in 9.4% (37/391) in the third and fourth week, and in 5.9% (23/391) after more than 28 days. After the neonatal period, most of the patients followed a lactose-free diet, 94.2% (406/431). The majority of patients adhered to a relaxed diet (lactose free without further restrictions), in 64.3% (245/381) rather than a strict diet (lactose free and restriction of non-dairy sources) in 35.7% (136/381) (Additional file 5: Table S5).

## Discussion

The aim of this descriptive study was to delineate the natural history of patients with a residual GALT activity of ≤10% and/or GALT severe disease-causing mutations based on the largest cohort studied so far (*n* = 509) from many countries with different genetic backgrounds. Our data confirm that most patients experience neonatal illness, and that despite the diet, they develop brain and gonadal impairments and are at risk for a lower BMD.

### Neonatal illness

This analysis in a large study population is in agreement with earlier descriptions of frequent occurrence of liver damage with bleeding diathesis, and infection in the neonatal period [17, 30]. A reported lower enzyme activity was related to a higher rate of neonatal illness. Diagnosis through NBS was associated with a more favorable neonatal presentation. These patients were younger at diagnosis and diet was more often introduced in the first week of life. The positive effect of early dietary treatment on neonatal complications has been known for a long time [31, 32] and these results corroborate this finding.

### Neurological, cognitive and behavioral complications

There is a high occurrence of brain impairments, including developmental and language delay, neurological complications, language and speech disorders and mental and behavioral problems. The most frequently seen neurological symptom was tremor, in 31.0% (104/336) with a lower occurrence than in previous reports with smaller cohorts [30, 33]. The prevalence of other neurological symptoms (general motor abnormality ataxia, seizures and dystonia) was in line with earlier findings [30, 33]. In this large dataset, we were able to also assess the time of onset of the complications. Tremor was more often seen after the second decade of life, but also between the first year and pre-school age and between pre-school age and the second decade.

Importantly, the occurrence of mental and behavioral problems was not different from the general population. The most frequently reported were anxiety disorder and general behavioral problems. Other complications included depression, ADHD and ASD. Behavioral problems were more likely to occur before the second decade, whereas mental (psychiatric) problems, such as depression and anxiety disorder were more predominant after the second decade. Anxiety disorders were common in all age categories. In previous reports, based on smaller patient cohorts, the occurrence of anxiety disorders in adults was reported to be higher [30, 33]. This difference could be due to the age distribution in our data set.

Interestingly, patients with a relaxed diet (lactose free without further restrictions) less often developed neurological complications. Previous studies suggested that a more relaxed diet did not seem to be associated with a more severe clinical outcome [2, 34–36]. A recent study in a large patient cohort (*n* = 231) reported that the rigor of non-dairy galactose restriction in early childhood does not associate with severity of long-term outcomes growth, adaptive behaviors, receipt of speech therapy, receipt of educational services and ovarian function [37]. Moderate liberalization of galactose intake (suggested due to galactose’s importance for glycosylation of glycoproteins and glycolipids) has been shown to improve IgG glycosylation in a small subset of pediatric and adult patients [38, 39]. It is possible like other inborn errors of metabolism requiring substrate precursors that a minimum amount of exogenous dietary galactose is necessary for all CG patients. Our results support the moderate liberalization of diet that is recommended nowadays [40–42].

### Gonadal complications

Ovarian damage was reported in a vast majority of female patients. Hypergonadotropic hypogonadism in women with CG was first described in 1979 [18]. Subsequently, POI was broadly recognized and represents a very burdensome complication for the patients and their families. The occurrence of POI in this study was comparable to previous figures [17, 30, 43, 44]. A high percentage of women with POI was taking HRT to supplement hormonal insufficiency, reflecting an appropriate follow-up.

In a previous study by van Erven et al. (2017), 29.6% of the patients tried to conceive, and successful pregnancy was achieved in 42.9% [20]. Our findings reiterate the need for adjustment in counseling of these women regarding fertility and reproduction. In the past, fertility counseling has been discouraging and many women abandoned trying to conceive. This data strengthens the notion that, in reproduction counseling subfertility rather than infertility should be acknowledged which carries implications for the patients when considering fertility preservation and family planning including contraception if pregnancy is not desired.

In male subjects, the prevalence of cryptorchidism in this larger study is less than what was previously described [45, 46], but still higher than in the general population (1.0%). Pubertal delay in males is not different compared to the general population [47]. This is in line with male gonads not being clinically significantly affected. Only a small percentage of male patients fathered a child. This could possibly be explained by the social difficulties [34] and/or delayed psychosexual and social development in young adult men with galactosemia [48].

### Bone health

A reduced BMD was first described in 1993 [19]. Thereafter, several studies confirmed this feature in patients with CG [30, 49–52]. Hitherto, it is not clear whether this is secondary to the restricted diet, a primary intrinsic disease effect or a combination of both. In this cohort we also found reduced BMD in accordance with previous studies. The majority of patients received vitamin D and calcium supplementation to meet daily recommended requirements. Patients with a low BMD were mostly taking these supplements, but despite the supplementation BMD was still lower. These findings are in line with the experience in treating these patients, BMD might improve but not normalize when given supplements. The number of fractures in this cohort was not higher than in the general population [53]. It should be noted, however, that the median age in this cohort was relatively young.

### Predictive factors

The development of long-term complications seems to be associated with NBS, age at onset of dietary treatment, strictness of the galactose-restricted diet, GALT enzyme activity and genotype. We found that use of NBS was associated with a lower rate of neurological symptoms. Since 2005, several countries have implemented NBS for CG. One exception is Ireland, where they perform an NBS for galactosemia since 1972 because of the high prevalence [2]. Evaluation of the effectiveness of NBS in the Netherlands showed a benefit of NBS in preventing critical illness [54]. Our data support this concept and can be taken into consideration by decision-makers for implementation of NBS for CG in national programs. Enzyme activity ≤ 1% was associated with more mental and behavioral problems. Homozygosity for p.Gln188Arg and a strict diet were both associated with a higher rate of neurological complications and POI. Peak neonatal erythrocyte Gal-1-P level showed no correlation with any individual outcome measures. Mean erythrocyte Gal-1-P was not recorded in our registry. In many centers this parameter is not used for the regular follow-up once an individual baseline has been established.

### Study limitations

Although a large cohort has been studied, there are limitations to be considered. First, this is a retrospective observational study and not all patients had been followed in a standardized systematic manner, in contrast with the cohort study by Waisbren et al. [30], where patients received a standardized evaluation, e.g. examination by a neurologist, endocrine testing and psychological evaluation. In this registry study, not all patients had received a neurological evaluation by a neurologist, and the assessment of tremor, ataxia, dystonia e.g. might not be fully accurate. Furthermore, the whole age range is included with median of 18 years (range 0–65 years), implying that for several variables (e.g. POI) data could not be available. All patient data derived from medical history files, and not all data was complete and available for collection. This led to limitations in analyzing possible associations between several factors and the outcomes. Nevertheless, the registry is still open, and we expect more data entry from numerous other centers in the upcoming years to allow multivariate analysis.

## Conclusions

In summary, this study provides a description of the natural history of classic galactosemia based on a large data set. This study confirms that most affected patients experience neonatal illness, 79.8% (332/416) and, despite the diet, develop brain impairments in 85.0% (277/316), POI in 79.7% (118/148) and a lower BMD in 26.5% (76/287). NBS, age at onset of dietary treatment, strictness of the galactose-restricted diet, and GALT enzyme activity influence the clinical picture. Onset of diet in the first week, and diagnosis with NBS are related to a more favorable outcome. A strict diet, a GALT enzyme activity ≤ 1% and homozygosity for p.Gln188Arg were associated with a less favorable outcome.

## Patients and methods

### GalNet registry

In 2012, the international network for the galactosemias (GalNet) was established [24]. The GalNet has developed and implemented an international web-based patient registry, which currently includes centers from several European countries, Israel and the United States (Additional file 6: Table S6 and Additional file 7: Table S7, participating centers and countries). It was established in accordance with Good Clinical Practice and is in compliance with General Data Protection Regulation. Data was collected from medical files and coded before entered on the encrypted password protected registry. Only the principal investigator (PI) had access to the encrypted code corresponding to the specific patient. The registry contains information on patients with any type of galactosemia: classic galactose-1-phosphate uridylyltransferase deficiency (GALT deficiency, OMIM #230400), galactokinase deficiency (GALK1, OMIM #230200) and UDP-galactose-4-epimerase deficiency (GALE, OMIM #230350). The coordinating center (Maastricht University Medical Center+ (MUMC+)) has developed the registry, adapted from a Harvard University-based RedCap system (https://ecrf.ctcm.nl/macro/). The MACRO software is used, installed and configured according to the Elsevier manual on a server of the MUMC+ (https://www.elsevier.com/about/policies/privacy-principles/gdpr). The study was approved by the local ethics committee of the coordinating center, application number METC 13–4-121.6/ab, and subsequently approved by participating partners. PIs from contributing centers submitted the registry proposal to their local institutions for ethical approval according to national laws and regulations. A letter of agreement was signed by participating centers for the use of data. Following approval, training was provided to the responsible PIs by the coordinating center. This training included explanation on the content of the electronic case report (eCRFs) and how to enter data. Participants were approached by their treating physicians to participate in the registry and written consent was obtained from all patients or their authorized representatives prior to data entry. Data curation by the coordinating center was performed regularly. Overviews of missing data were provided, and PIs were contacted to complete datasets.

### Inclusion and exclusion criteria

Data derived from 15 countries and 32 centers, were acquired between December 2014 and July 2018. For this study, only patients with confirmed classic and variant galactosemia (diagnosed by a residual GALT activity of ≤10% and/or GALT pathogenic disease-causing mutations) were included for analysis. Patients with GALK1 or GALE deficiency were not included. The total number of patients included for this analysis was 509 (Additional file 6: Table S6 and Additional file 7: Table S7, participating centers and countries).

### Content GalNet registry

Data entry was based on an eCRF using a set of agreed parameters developed by the GalNet experts. A Harvard University-based RedCap system was used to generate a comprehensive platform to electronically capture data information on subjects with one of the galactosemias. This system was shared and adjusted at the MUMC+ with input from European experts to develop an ease of use system that could be used around the world. The registry consists of seven eCRFs (1. Demographics; 2. Neonatal information; 3. General follow-up; 4. Brain follow-up; 5. Gonads and reproduction follow-up; 6. Bone health follow-up; 7. Diet) and contains a user’s manual with explanations on the different variables (Additional file 8).

### Statistical analysis

Data for analysis was exported from the original database in MACRO to SPSS (IBM SPSS Statistics version 23). Patient data included in the registry prior to the 31st of July 2018 were included in the analysis. Descriptive analysis showed medians and ranges for continuous variables and frequencies and percentages for categorical variables. Differences between groups were analyzed using Fisher’s exact test for categorical variables. All clinical outcomes have been classified in two categorical groups (presence vs. absence of outcome) to assess the association of a certain variable (present vs. absent) with a clinical outcome, using a Fisher’s exact test for categorical variables. Odds ratios and 95% confidence intervals with *p* values are presented. Logistic regression was performed if the total number of samples was sufficient. A *p* value < 0.05 is considered statistically significant. Our analysis accounted for missing data due to difficulties in retrieving historical data. In addition, for some patients some variables were not yet known due to a young age. When the number of missing data is > 10%, as is the case for this registry, the results of subsequent statistical analyses may be biased [55]. Patterns of missing variables were traceable or predictable from other variables in the dataset. Methods to handle the missing observations included performing available case analysis (for the descriptive analysis) and complete case analysis (for associations analysis, odds ratios and Fisher’s exact test). The number of available data per variable is called the valid number. Valid numbers are shown in the text, Tables 1, 2, 3 and 4, as n/valid n.

## Additional files

Additional file 1: **Table S1.** International Standard Classification of Education (ISCED). ISCED 0 Early childhood education; ISCED 1 Primary education; ISCED 2 Lower secondary education; ISCED 3 Upper secondary education; ISCED 4 Post-secondary non-tertiary education; ISCED 5 Short-cycle tertiary education; ISCED 6 Bachelor’s or equivalent level; ISCED 7 Master’s or equivalent level; ISCED 8 Doctoral or equivalent level. ^*^Included patients have all completed education. ^**^82.9% of the siblings are still in education. (PDF 173 kb)

Additional file 2: **Table S2.** Social or occupational classification: Registrar General’s Social Class (RGSC). (PDF 71 kb)

Additional file 3: **Table S3.** Bone health. BMD was measured by dual energy x-ray photometry (DXA) using T- or Z-scores of the lumbar spine, depending on age and gender. T-scores were used in post-menopausal women and men ≥ 50 years. Z-scores, considering age, gender, and ethnicity, were used in pre-menopausal women and men < 50 years. A low BMD was classified as BMD T-score ≤- 1.0 standard deviation (SD) or a BDM Z-score ≤- 2.0 SD. Median BMD Z-score in studied population was - 0.8 SD, range - 5.1 to 4.0 SD. Median BMD T-score in studied population was - 1.1 SD, range - 4.0 to 4.3 SD. ^1^ 65.8% was female. ^2^Median age of patients who reported fractures was 24 years, range 6 to 59 years. A low BMD was present in 23.8% (5/21) of the patients with fractures, 61.1% of patients with a fracture was male. ^3^The World Health Organization (WHO) recommends 60 minutes of moderate- to vigorous-intensity physical activity per day for children and 150 minutes per week for adults. (PDF 71 kb)

Additional file 4: **Table S4.** Growth and cataracts. ^*^Median 29.5 years, range 18 to 41 years. (PDF 54 kb)

Additional file 5: **Table S5.** Dietary treatment. ^*^We have no information on siblings’ relations. ^1^A strict diet was defined as lactose free and restriction of non-dairy sources (at least one of the following: galactosides, fruit and vegetables and/or nucleoproteins), with an estimated intake of galactose < 20 mg/day. ^2^A relaxed diet was defined as lactose free without further restrictions with an estimated galactose intake <100 mg/day. (PDF 73 kb)

Additional file 6: **Table S6.** Participating countries and respective center(s). Total number of included patients for analysis: 509. (PDF 241 kb)

Additional file 7: **Table S7.** Participating countries. Total number of included patients for analysis: 509. (PDF 51 kb)

Additional file 8: Electronic Case Report Forms (eCRFs). (PDF 344 kb)

## Abbreviations

ADHD: Attention deficit hyperactivity disorder
ASD: Autism spectrum disorder
BMD: Bone mineral density
CG: Classic galactosemia
eCRF: Electronic case report form
Gal-1-P: Galactose-1-phosphate
GALE: UDP-galactose epimerase
GALK1: Galactokinase1
GalNet: Galactosemia network
GALT: Galactose-1-phosphate uridylyltransferase
HRT: Hormone replacement therapy
ISCED: International Standard Classification of Education
NBS: Newborn screening
PI: Principal investigator
POI: Primary ovarian insufficiency
RGSC: Registrar General’s Social Class
WHO: World Health Organization
