# Supplementary material for: The natural history of classic galactosemia: lessons from the GalNet registry
Source: Orphanet J Rare Dis. 2019 Apr 27;14:86. doi: 10.1186/s13023-019-1047-z (PMC6486996; doi:10.1186/s13023-019-1047-z)
Supplement: Supplementary file 1 — Table S1. International Standard Classification of Education (ISCED). ISCED 0 Early childhood education; ISCED 1 Primary education; ISCED 2 Lower secondary education; ISCED 3 Upper secondary education; ISCED 4 Post-secondary non-tertiary education; ISCED 5 Short-cycle tertiary education; ISCED 6 Bachelor’s or equivalent level; ISCED 7 Master’s or equivalent level; ISCED 8 Doctoral or equivalent level. *Included patients have all completed education. **82.9% of the siblings are still in education. (PDF 173 kb) [file 13023_2019_1047_MOESM1_ESM.pdf]

Table S4

Table S4. International Standard Classification of Education (ISCED). ISCED 0 Early childhood education; ISCED 1 Primary education; ISCED 2 Lower secondary education; ISCED 3 Upper secondary education; ISCED 4 Post-secondary non-tertiary education; ISCED 5 Short-cycle tertiary education; ISCED 6 Bachelor's or equivalent level; ISCED 7 Master's or equivalent level; ISCED 8 Doctoral or equivalent level.

	Patient *			Father			Mother			Non galactosemia sibling**		
	**n**	**valid n**	**%**	**n**	**valid n**	**%**	**n**		**%**	**n**	**valid n**	**%**
		177			192			192			70	
**ISCED 0-3**	73		41.2	51		26.6	50		26.0	58		82.9
**ISCED 4-6**	75		42.4	59		30.7	83		43.2	12		17.1
**ISCED 6-9**	29		16.4	82		42.7	59		30.7			
		161									103	
**Regular education**	119		73.9							100		97.1
**Special education**	42		26.1							3		2.9

* Included patients have all completed education.
** 82.9% of the siblings are still in education.
