# Supplementary material for: The natural history of classic galactosemia: lessons from the GalNet registry
Source: Orphanet J Rare Dis. 2019 Apr 27;14:86. doi: 10.1186/s13023-019-1047-z (PMC6486996; doi:10.1186/s13023-019-1047-z)
Supplement: Supplementary file 2 — Table S2. Social or occupational classification: Registrar General’s Social Class (RGSC). (PDF 71 kb) [file 13023_2019_1047_MOESM2_ESM.pdf]

Table S5

Table S5. Social or occupational classification: Registrar General's Social Class (RGSC).

	Patient			Father			Mother		
	**n**	**valid n**	**%**	**n**	**valid n**	**%**	**n**	**valid n**	**%**
		149			200			209	
**Professional**	5		3.4	25		12.5	24		11.5
**Managerial and technical**	9		6.0	46		23.0	26		12.4
**Skilled**	33		22.1	76		38.0	78		37.3
Manual	10		30.3	45		59.2	14		17.9
Non-Manual	22		66.7	31		40.8	60		76.9
**Partly skilled**	34		22.8	20		10.0	25		12.0
**Unskilled**	68		45.6	33		16.5	56		26.8
