# Supplementary material for: The natural history of classic galactosemia: lessons from the GalNet registry
Source: Orphanet J Rare Dis. 2019 Apr 27;14:86. doi: 10.1186/s13023-019-1047-z (PMC6486996; doi:10.1186/s13023-019-1047-z)
Supplement: Supplementary file 3 — Table S3. Bone health. BMD was measured by dual energy x-ray photometry (DXA) using T- or Z-scores of the lumbar spine, depending on age and gender. T-scores were used in post-menopausal women and men ≥ 50 years. Z-scores, considering age, gender, and ethnicity, were used in pre-menopausal women and men < 50 years. A low BMD was classified as BMD T-score ≤- 1.0 standard deviation (SD) or a BDM Z-score ≤- 2.0 SD. Median BMD Z-score in studied population was - 0.8 SD, range - 5.1 to 4.0 SD. Median BMD T-score in studied population was - 1.1 SD, range - 4.0 to 4.3 SD. 1 65.8% was female. 2Median age of patients who reported fractures was 24 years, range 6 to 59 years. A low BMD was present in 23.8% (5/21) of the patients with fractures, 61.1% of patients with a fracture was male. 3The World Health Organization (WHO) recommends 60 minutes of moderate- to vigorous-intensity physical activity per day for children and 150 minutes per week for adults. (PDF 71 kb) [file 13023_2019_1047_MOESM3_ESM.pdf]

Table S1

Table S1. Bone health. BMD was measured by dual energy x-ray photometry (DXA) using T- or Z-scores of the lumbar spine, depending on age and gender. T-scores were used in post-menopausal women and men ≥ 50 years. Z-scores, considering age, gender, and ethnicity, were used in pre-menopausal women and men < 50 years. A low BMD was classified as BMD T-score ≤ -1.0 standard deviation (SD) or a BDM Z-score ≤ -2.0 SD.
Median BMD Z-score in studied population was -0.8 SD, range -5.1 to 4.0 SD
Median BMD T-score in studied population was -1.1 SD, range -4.0 to 4.3 SD

	n	valid n	%
**Low BMD**[1]	76	287	26.5
**Fractures**[2]	21	213	9.9
**Vitamin D level**		200	
**Normal** (> 75 nmol/l)	77		38.5
**Insufficiency** (50-75 nmol/l)	70		35.0
**Deficiency** (0-49 nmol/l)	53		26.5
**Vitamin D deficiency + calcium supplement**	35	46	76.1
**Vitamin D deficiency + vitamin D supplement**	38	47	80.9
**Calcium supplement**	281	412	68.2
**Vitamin D supplement**	288	405	71.1
**Adequate physical activity** according to WHO recommendation[3]	140	186	75.3

[1] 64.9 % was female.
[2] Median age of patients who reported fractures was 24 years, range 6 to 59 years. A low BMD was present in 23.8% (5/21) of the patients with fractures, 61.1% of patients with a fracture was male.
[3] The World Health Organization (WHO) recommends 60 minutes of moderate- to vigorous-intensity physical activity per day for children and 150 minutes per week for adults.
