# Supplementary material for: The natural history of classic galactosemia: lessons from the GalNet registry
Source: Orphanet J Rare Dis. 2019 Apr 27;14:86. doi: 10.1186/s13023-019-1047-z (PMC6486996; doi:10.1186/s13023-019-1047-z)
Supplement: Supplementary file 4 — Table S4. Growth and cataracts. *Median 29.5 years, range 18 to 41 years. (PDF 54 kb) [file 13023_2019_1047_MOESM4_ESM.pdf]

Table S2

Table S2. Growth and cataracts.

	n	valid n	%
**Short stature** (outside TH range)	38	374	10.2
**Cataract in the neonatal period**	68	264	25.8
**Persistent cataract in childhood**	20	44	45.5
**Persistent cataract in adulthood**	5	20	25.0
**Cataract reported in childhood**	22	238	9.2
**Cataract reported in adulthood***	10	89	11.2

* Median 29.5 years, range 18 to 41 years
