# Supplementary material for: The natural history of classic galactosemia: lessons from the GalNet registry
Source: Orphanet J Rare Dis. 2019 Apr 27;14:86. doi: 10.1186/s13023-019-1047-z (PMC6486996; doi:10.1186/s13023-019-1047-z)
Supplement: Supplementary file 5 — Table S5. Dietary treatment. *We have no information on siblings’ relations. 1A strict diet was defined as lactose free and restriction of non-dairy sources (at least one of the following: galactosides, fruit and vegetables and/or nucleoproteins), with an estimated intake of galactose < 20 mg/day. 2A relaxed diet was defined as lactose free without further restrictions with an estimated galactose intake <100 mg/day. (PDF 73 kb) [file 13023_2019_1047_MOESM5_ESM.pdf]

Table S3

Table S3. Dietary treatment.

	n	valid n	%
**Infant formula**		394	
Soy	302		76.6
Elemental formula	50		12.7
Other formula or combination	42		10.7
**Age onset diet**		392	
day 0-1*	65		16.6
day 2-7	133		33.9
day 8-14	134		34.2
day 15-28	37		9.4
>28 days	23		5.9
**Lactose free diet**	406	431	94.2
**Not on lactose free diet**	25	431	5.8
**Restrict galactosides**	109	416	26.2
**Restrict fruit and vegetables**	95	429	22.1
**Restrict nucleoproteins**	113	412	27.4
**Cheese allowed** (mature, old cheeses)	275	384	71.6
**Strict diet**[1]	136	381	35.7
**Relaxed diet**[2]	245	381	64.3

* We have no information on siblings' relations.

[1] A strict diet was defined as lactose free and restriction of non-dairy sources (at least one of the following: galactosides, fruit and vegetables and/or nucleoproteins), with an estimated intake of galactose < 20 mg/day.

[2] A relaxed diet was defined as lactose free without further restrictions, with an estimated galactose intake <100 mg/day.
