# Supplementary material for: The natural history of classic galactosemia: lessons from the GalNet registry
Source: Orphanet J Rare Dis. 2019 Apr 27;14:86. doi: 10.1186/s13023-019-1047-z (PMC6486996; doi:10.1186/s13023-019-1047-z)
Supplement: Supplementary file 6 — Table S6. Participating countries and respective center(s). Total number of included patients for analysis: 509. (PDF 241 kb) [file 13023_2019_1047_MOESM6_ESM.pdf]

Table S6

Table S6. Participating countries and respective center(s).

Country and respective center(s)	N. of patients
**Austria**	
Medizinische Universität Wien Vienna	12
Medical University Innsbruck	7
University Children’s Hospital, Parcelsus Medical University, Salzburg	3
**Belgium**	
University Hospital Antwerpen	5
University Hospital Leuven	5
**Croatia**	
Klinički bolnički centar Zagreb	7
**Estonia**	
Tartu University Hospital	8
**France**	
Hôpital Antoine Béclère, Clamart	27
**Germany**	
Uniklinik RWTH Aachen	27
**Ireland**	
National Centre for Inherited Metabolic Disorders Dublin (Temple St Childrens Univeristy Hospital and Mater Misericordiae University Hospital)	70
**Israel**	
Sheba Medical Center Ramat Gan	2
**Lithuania**	
Vilniaus universiteto ligoninės Santaros klinikos Santariškės	1
**Netherlands**	
Erasmus Medical Center Rotterdam	31
Maastricht University Medical Center	30
Amsterdam Medical Center	28
University Medical Center Nijmegen	14
University Medical Center Groningen	8
VU Medical Center Amsterdam	3
University Medical Center Utrecht	1
**Portugal**	
Hospital Santa Maria Lisboa	13
**Switzerland**	
Inselspital, University Hospital Bern	24
CHUV, University Hospital Lausanne	3
University Hospital Zurich	4
University Children‘s Hospital Zurich	4
**Spain**	
Hospital Clinic de Barcelona	12
Complejo Hospitalario Universitario de Santiago	3

Country and respective center(s)	N. of patients
**United Kingdom**	
Salford Royal NHS Foundation Trust London	41
University College London	13
Great Ormond Street Hospital London	11
Evelina Children's Hospital St Thomas' Hospital Westminster Bridge Road London	2
University Hospital Birmingham	5
**United States**	
Boston Children’s Hospital	85

Total number of included patients for analysis: 509
