# Supplementary material for: The natural history of classic galactosemia: lessons from the GalNet registry
Source: Orphanet J Rare Dis. 2019 Apr 27;14:86. doi: 10.1186/s13023-019-1047-z (PMC6486996; doi:10.1186/s13023-019-1047-z)
Supplement: Supplementary file 7 — Table S7. Participating countries. Total number of included patients for analysis: 509. (PDF 51 kb) [file 13023_2019_1047_MOESM7_ESM.pdf]

Table S7

Table S7. Participating countries.

Country	N. of patients
Austria	22
Belgium	10
Croatia	7
Estonia	8
France	27
Germany	27
Ireland	70
Israel	2
Lithuania	1
Netherlands	115
Portugal	13
Switzerland	35
Spain	15
United Kingdom	72
United States	85

Total number of included patients for analysis: 509
