# Supplementary material for: The natural history of classic galactosemia: lessons from the GalNet registry
Source: Orphanet J Rare Dis. 2019 Apr 27;14:86. doi: 10.1186/s13023-019-1047-z (PMC6486996; doi:10.1186/s13023-019-1047-z)
Supplement: Supplementary file 8 — Electronic Case Report Forms (eCRFs). (PDF 344 kb) [file 13023_2019_1047_MOESM8_ESM.pdf]

Galactosemia Network

ctcm clinical trial center maastricht

Registry manual

The required information to be entered in the registry is summarized below. It is advised to have these data available before beginning with the data entering process.

- Date of birth
- Gender
- Mutation(s)
- Enzyme activity and method used for measurement
- Level of education and social an occupational classification (patient, parents an sibling(s))

Level of education (ISCED 2011) explained below:

ISCED 0 Early childhood education

ISCED 1 Primary education

Fundamental skills in reading, writing and mathematics.
Typically lasts 6 years from age 5-7.
Preparation for ISCED level 2.

Primary education, elementary education or basic education (stage 1 or lower grades if an education system has one program that spans ISCED levels 1 and 2).

ISCED 2 Lower secondary education

More subject oriented curriculum, introducing theoretical concepts across a broad range of subjects. Typically lasts from age 10-13 to 14-16 years. Preparation for ISCED level 3.

Secondary school (stage one/lower grades if there is one program that spans ISCED levels 2 and 3), junior secondary school, middle school or junior high school.

If a program spans ISCED levels 1 and 2, the terms elementary school or basic school (stage 2/upper grades) are often used.

ISCED 3 Upper secondary education

Designed to complete secondary education. More differentiated, with an increased range of options and streams available.
Typically lasts from age 14-16 to 17-18 years.
Possibly direct access to ISCED level 4 and/or level 5, 6 or 7.

Secondary school (stage two/upper grades), senior secondary school, or (senior) high school.

ISCED 4 Post-secondary non-tertiary education

Non-tertiary qualifications required for progression to tertiary education (ISCED level 5, 6 or 7) or employment.

Technician diploma, primary professional education, or préparation aux carrières administratives.

ISCED 5 Short-cycle tertiary education

Practically-based, occupationally specific and preparing student to enter the labor market or provide a pathway to other tertiary programs.
Requires successful completion of ISCED level 3 or 4 with access to tertiary education.

(Higher) technical education, community college education, technician or advanced/higher vocational training, associate degree, or bac +2.

ISCED 6 Bachelor's or equivalent level

Academic and/or professional knowledge, skills and competencies, leading to a first degree or equivalent qualification.

Requires successful completion of ISCED level 3 or 4 with access to tertiary education or sometimes after completion of ISCED level 5.

Bachelor's program, license, first university cycle.

ISCED Level 7 Master's or equivalent level

Advanced academic and/or professional knowledge, skills and competencies, leading to a second degree or equivalent qualification.
Requires successful completion of an ISCED level 6 or 7 program.

Master programs or magister.

ISCED Level 8 Doctoral or equivalent level

Leading to an advanced research qualification.
Requires successful completion of specific ISCED level 7 programs.

PhD, DPhil, D.Lit, D.Sc, LL.D, Doctorate or similar terms.

The complete INTERNATIONAL STANDARD CLASSIFICATION OF EDUCATION 2011 can b
from http://www.uis.unesco.org/Education/Pages/international-standard-classification-of-educ

- Neonatal data

Date of diet introduction

Neonatal illness

Encephalopathy

PTT/APTT/ALAT/ASAT/glucose values

If sepsis blood culture data GAL

- 1 - P (peak) concentration

- Date general follow up
- Length, weight, head circumference
- Ophthalmology data
- Brain follow up

Imaging (if available)

Data on development and neurological assessment

- Female - gonads

Spontaneous puberty

Induced

Hormone measurements

Pregnancy / children, if yes birth date of children

- Male - gonads

Cryptorchidism?

- Bones

DEXA: Lumbar spine Z-score or T-score

Fractures

Vit D (25-hydroxy D3) measurement

- Diet

Infant formula used

Recommended diet items

Ca supplements

Ca intake

Regarding follow-up, different evaluations at different time points can be filled in, by creating an extra eform. To do so, answer the question with 'yes' at the bottom of the page.

Demographics

Demographics: Patient

If the language setting of your computer is set to English, please use a 'dot' to enter the digits after decimals.

If the language setting of your computer is set to Dutch, please use a 'comma' to enter the digits after decimals.

Patient ID

[ ] *(XXXX)*

Gender

◎ Male

◎ Female

Ethnicity all sites

If other, define:

Galactosemia

Kind of galactosemia

GALT

GALT gene mutation

If other, define:

GALT enzyme activity

[ ] *(%)* ◎ Unknown

◎ Below detection limit

Detection limit

(%)

Measurement method GALT enzyme activity

◎ Radioactive
◎ Fluorescence enzyme coupled reaction (newborn screening public health laboratory)
◎ Fluorescence enzyme coupled reaction (biochemical genetics diagnostic laboratory)
◎ LC-MS/MS
◎ HPLC (High Pressure Liquid Chromatography)
◎ Unknown

GALE

GALE gene mutation

... ◎ Unknown

GALE enzyme activity (%)

◎ Unknown

Detection limit

◎ Below detection limit

(%)

GALK

GALK gene mutation

... ◎ Unknown

GALK enzyme activity (%)

◎ Unknown

Detection limit

◎ Below detection limit

(%)

Level of education

Age at collection of following data

(years) ...

Please specify highest level of education (please answer questions below as well)

◎ Unknown

☐ Not applicable

Highest level of education if patient is younger than 18 years old

◎ Regular education
◎ Special education

Level of education (ISCED 2011), see registry manual for detailed information

◎ ISCED 0 Early childhood education
◎ ISCED 1 Primary education
◎ ISCED 2 Lower secondary education
◎ ISCED 3 Upper secondary education
◎ ISCED 4 Post-secondary non tertiary education
◎ ISCED 5 Short-cycle tertiary education
◎ ISCED 6 Bachelor's or equivalent level
◎ ISCED 7 Master's or equivalent level
◎ ISCED 8 Doctoral or equivalent level
◎ Not applicable

Occupation/profession

Please specify current occupation/profession (please answer questions below as well)

☐ Education in progress
◎ Unknown
◎ Not applicable

Social or occupational classification if patient is older than 18 years old (RGSC)

◎ I - Professional etc occupations - e.g. professors, doctors, laywers,
◎ II - Managerial and Technical occupations - self-employed, proprietors SME; manager, engine
◎ III - Skilled occupations
◎ IV - Partly-skilled occupations - call center employees
◎ V - Unskilled occupations - incl. unemployed, ancillary position

RGSC 3

◎ III: non-manual - e.g. IT-professions, health care professionals
◎ III: manual - e.g. mechanic

Demographics: Family of patient

Father

Please specify highest level of education (please answer questions below as well)

◎ Unknown

Level of education father (ISCED 2011), see registry manual for detailed information

◎ ISCED 0 Early childhood education
◎ ISCED 1 Primary education
◎ ISCED 2 Lower secondary education
◎ ISCED 3 Upper secondary education
◎ ISCED 4 Post-secondary non-tertiary education
◎ ISCED 5 Short-cycle tertiary education
◎ ISCED 6 Bachelor's or equivalent level
◎ ISCED 7 Master's or equivalent level
◎ ISCED 8 Doctoral or equivalent level

Occupation/profession

Please specify current occupation/profession (please answer questions below as well)

◎ Unknown

Social or occupational classification (RGSC)

◎ I - Professional etc occupations - e.g. professors, doctors, laywers,
◎ II - Managerial and Technical occupations - self-employed, proprietors SME; manager, engine
◎ III - Skilled occupations
◎ IV - Partly-skilled occupations - call center employees
◎ V - Unskilled occupations - incl. unemployed, ancillary position

RGSC 3

◎ III: non-manual - e.g. IT-professions, health care professionals
◎ III: manual - e.g. mechanic

Mother

Please specify highest level of education (please answer questions below as well)

◎ Unknown

Level of education mother (ISCED 2011), see registry manual for detailed information

◎ ISCED 0 Early childhood education

◎ ISCED 1 Primary education

◎ ISCED 2 Lower secondary education

◎ ISCED 3 Upper secondary education

◎ ISCED 4 Post-secondary non-tertiary education

◎ ISCED 5 Short-cycle tertiary education

◎ ISCED 6 Bachelor's or equivalent level

◎ ISCED 7 Master's or equivalent level

◎ ISCED 8 Doctoral or equivalent level

Occupation/profession

Please specify current occupation/profession (please answer questions below as well)

◎ Unknown

Social or occupational classification (RGSC)

◎ I - Professional etc occupations - e.g. professors, doctors, laywers,

◎ II - Managerial and Technical occupations - self-employed, proprietors SME; manager, engine

◎ III - Skilled occupations

◎ IV - Partly-skilled occupations - call center employees

◎ V - Unskilled occupations - incl. unemployed, ancillary position

RGSC 3

◎ III: non-manual - e.g. IT-professions, health care professionals

◎ III: manual - e.g. mechanic

Sibling(s)

Number of siblings

◎ Unknown

Please specify highest level of education per sibling (please answer questions below as well)

◎ Unknown

Please specify current occupation/profession per sibling (please answer questions below as wel

☐ Education in progress

☐ Unknown

Number	Healthy	Galactosemia	Age	Highest level of education < 18
...	Yes / No	Yes / No	<18 / >=18	
...	Yes / No	Yes / No	<18 / >=18	
...	Yes / No	Yes / No	<18 / >=18	
...	Yes / No	Yes / No	<18 / >=18	
...	Yes / No	Yes / No	<18 / >=18	

Date of data collection
(dd/mm/yyyy)

Galactosemia Network

clinical trial center maastricht

Patient number

Gender

Neonatal information

Neonatal signs and symptoms

Age at onset of galactose restricted/free diet

Years	Months	Weeks	Days

Diagnosed with newborn screening

Acute neonatal illness

Encephalopathy in newborn period
(altered mental state: depressed consciousness with or without neurological signs)

Cataract in newborn period

Bleeding diathesis in newborn period
(abnormal PT APTT)

Infection in newborn period
(clinical signs of infection/sepsis)

Positive blood culture

Positive blood culture remark

Neonatal clinical chemistry

Elevated liver enzymes in the newborn period
(ALT, AST > 30 U/L)

Hypoglycemia in newborn period (<2.6 mmol/L)

Neonatal additional metabolic testing

Peak galactose-1-phosphate in newborn period *(Please use comma for decimal)*

(μmol/g Hb, XXX,XX)

(mg %, XXX)

If other unit than above, please specify (including unit)

◎ Not done

◎ Unknown

Date of follow up
(dd/mm/yyyy)

Galactosemia Network

Patient number

Gender

General follow-up

Did a long term follow up take place?

◎ Yes

◎ No

Date follow up

(dd/mm/yyyy)

Age

(years)

Head circumference (cm)

(XX,X) ◎ Unknown

Height (cm) *(Please use comma for decimal)*

(XXX,X) ◎ Unknown

Short stature (outside TH range)

Weight (kg)

(XXX,X)

◎ Unknown

Weight (pounds)

(XXX,X)

◎ Unknown

Lenticular changes/opacifications in childhood

Lenticular changes/opacifications adulthood (18-50)

Remarks (optional)

Open another eForm long term follow-up?

◎ **No**

◎ **Yes**

Date of data collection
(dd/mm/yyyy)

Galactosemia Network

ctcm clinical trial center maastricht

Patient number

Gender

Brain follow-up

Did a brain follow up take place?

◎ Yes

◎ No

Is data on brain imagining available?

If yes, describe

If other, describe

Developmental delay

Age of onset

Language delay

Age of onset

Impairment in grammar

Age of onset

General motor abnormality

Age of onset

Impairment in vocabulary

Age of onset

Speech defect

Age of onset

Verbal dyspraxia

Age of onset

Dysarthria

Age of onset

Ataxia

Age of onset

Chorea

Age of onset

Ballismus

Age of onset

Tremor

Age of onset

Dystonia

Age of onset

Athetosis

Age of onset

Seizures

Age of onset

Behavioral problems

Age of onset

ADHD

Age of onset

Diagnose of autism spectrum disorder

Age of onset

Depression

Age of onset

Anxiety disorder

Age of onset

Schizophrenia

Age of onset

Remarks (optional)

Open another eForm brain follow-up?

◎ **No**

◎ **Yes**

Date of data collection
(dd/mm/yyyy)

Galactosemia Network

ctcm clinical trial center maastricht

Patient number

Gender

Gonads and reproduction follow-up

Did a gonads and reproduction follow-up take place?

◎ Yes

◎ No

Delayed puberty
(Boys: lack of increase in testicle size by age 14
Girls: lack of breast development by age 13)

Children (only biological children)

Cryptorchidism

Spontaneous puberty

Age of onset

(years)

Induced puberty

Age of onset

(years)

Hormone replacement therapy
(beyond induction of puberty)

Age of onset

(years)

Primary ovarian insufficiency (POI)

Women < 40 years, >= 40 months amenorrhea
2 independent, more than 1 month apart FSH levels in the menopausal state

Gonads imaging

if yes, please specify imaging method used date of measurement and short summary o

Tried to conceive

Pregnancy

Spontaneous pregnancy

Assisted reproduction

Age of mother at first delivery

(Years)

How many children

(XX)

Open another eForm gonads and reproduction follow-up?

◎ **No**

◎ **Yes**

Date of data collection
(dd/mm/yyyy)

Galactosemia Network

ctcm clinical trial center maastricht

Patient number

Gender ...

Bone health follow-up

Did a bone health follow-up take place?

◎ Yes

◎ No

Date follow up

(dd/mm/yyyy)

Age

(years) ...

Bone mineral density lumbar spine (z-score) *(Please use comma for decimal)*

(+/- X,X)

Use Z-score:
1-for girls <18 yr or women >18 yr and premenopausal
2-for boys < 18yr and men <50 yr

Bone mineral density lumbar spine (t-score) *(Please use com*

(+/- X,X)

Use T-score:
1-for women>18 yr and postmenopausal
2-men >50 yr

Bone fractures

Vitamin D test performed?

If 25-hydroxy vit D test, please enter value in nmol/L *(Please use comma for decimal)*

(XX,X)

Other measurement for vitamin D ...

Does the patient use vitamin D or calcium supplements

Physical activity

The World Health Organization (WHO) recommends 60 minutes of moderate- to vigorous-intensity physical activity per day for children and 150 minutes per week for adults.

Open another eForm bone health follow-up?

◎ **No**

◎ **Yes**

Date of data collection (dd/mm/yyyy)

Galactosemia Network

ctcm clinical trial center maastricht

Patient number

Gender

Diet

Is data on diet available?

◎ Yes

◎ No

Which infant formula was used at diagnosis? *(Multiple options possible)*

◎ Soy

◎ Elemental

◎ Casein

◎ Hydrolysate with MCT

◎ Whey hydrolysate with MCT

◎ Whey hydrolysate with MCT

Is the recommended diet completely lactose free? *(No milk, yoghurt, butter or lactose as ingredient in food)*

Does the recommended diet restrict:

Galactosides (peas, beans, legumes, cocoa, soya)

Any fruit and vegetables (for their free and bound galactose)

Nucleoproteins (egg, liver, kidney)

Does the recommended diet allow a specified amount of galactose in the diet?

If yes: estimated amount in mg/day

(XXXX) ◎ Not calculated

Target calcium intake in mg/day Natural calcium intake in mg/day

(XXXX) (XXXX)

◎ Unknown ◎ Unknown

Calcium supplement Amount of calcium supplement (mg/day)

(XXXX,XX)

Vitamin D Amount of vitamin D

◎ microgram/day

◎ Unknown ◎ IU (International Unit)

Other vitamins or supplements If yes, specify

Is any type of cheese (derived from dairy milk) allowed?

If yes, specify type and name

Has the patient increased galactose intake independently? If yes, specify

Does the amount of daily allowed galactose increase with age?

If yes:

Do you purposely relax the diet in this patient?

[▽]

At what age do you relax the diet?

[ ] *(years)*

How much extra galactose do you aim to give?

[▽]

What is the reason for dietary relaxation in this patient?

◎ Patient choice

◎ New insights

◎ Other If other, specify [...]

Open another eForm diet?

◎ **No**

◎ **Yes**

Date of data collection (dd/mm/yyyy)

Galactosemia Network

ctcm clinical trial center maastricht

Patient number

Gender

...

Remarks

Remarks

Open another eForm Remarks?

◎ **No**

◎ **Yes**

Date of data collection (dd/mm/yyyy)

Galactosemia Network

ctcm clinical trial center maastricht

Patient number

Gender ...

End of Registry

End of registry

◎ Yes

◎ No

Date end of registry *(dd/mm/yyyy)*

Reason end of registry

◎ Patient died

◎ Loss to follow up

◎ Patient withdrew from study

◎ Other

Other reason ...
